# Dynamics of *V* *ibrio cholerae* abundance in Austrian saline lakes, assessed with quantitative solid‐phase cytometry

**DOI:** 10.1111/1462-2920.12861

**Published:** 2015-05-18

**Authors:** Sonja Schauer, Stefan Jakwerth, Rupert Bliem, Julia Baudart, Philippe Lebaron, Steliana Huhulescu, Michael Kundi, Alois Herzig, Andreas H. Farnleitner, Regina Sommer, Alexander Kirschner

**Affiliations:** ^1^ Institute for Hygiene and Applied ImmunologyMedical University ViennaVienna1090Austria; ^2^ Armament and Defence Technology AgencyNBC & Environmental Protection Technology DivisionVienna1090Austria; ^3^ UPMC Univ Paris 06USR 3579LBBMSorbonne UniversitésBanyuls‐sur‐Mer66650France; ^4^ CNRSLBBMUSR 3579Banyuls‐sur‐Mer66650France; ^5^ Austrian Agency for Health and Food Safety (AGES)Vienna1090Austria; ^6^ Institute for Environmental HealthMedical University of ViennaVienna1090Austria; ^7^ Biological Research Institute BurgenlandIllmitz7142Austria; ^8^ Research Group Environmental Microbiology and Molecular EcologyInstitute of Chemical EngineeringVienna University of TechnologyVienna1060Austria; ^9^ Interuniversity Cooperation Centre for Water and Health (ICC)ViennaAustria

## Abstract

In order to elucidate the main predictors of *V* *ibrio cholerae* dynamics and to estimate the risk of *V* *ibrio cholera*‐related diseases, a recently developed direct detection approach based on fluorescence *in situ* hybridization and solid‐phase cytometry (CARD‐FISH/SPC) was applied in comparison to cultivation for water samples from the lake Neusiedler See, Austria and three shallow alkaline lakes over a period of 20 months. *V* *ibrio cholerae* attached to crustacean zooplankton was quantified via FISH and epifluorescence microscopy. Concentrations obtained by CARD‐FISH/SPC were significantly higher than those obtained by culture in 2011, but were mostly of similar magnitude in 2012. Maximum cell numbers were 1.26 × 10^6^ *V* *. cholerae* per L in Neusiedler See and 7.59 × 10^7^ *V* *. cholerae* per L in the shallow alkaline lakes. Only on a few occasions during summer was the crustacean zooplankton the preferred habitat for *V* *. cholerae*. In winter, *V* *. cholerae* was not culturable but could be quantified at all sites with CARD‐FISH/SPC. Beside temperature, suspended solids, zooplankton and ammonium were the main predictors of *V* *. cholerae* abundance in Neusiedler See, while in the shallow alkaline lakes it was organic carbon, conductivity and phosphorus. Based on the obtained concentrations a first estimation of the health risk for visitors of the lake could be performed.

## Introduction

*Vibrio cholerae* is a ubiquitous member of microbial populations in moderately saline aquatic ecosystems (Colwell *et al*., 1977; Cottingham *et al*., 2003). *Vibrio cholerae* serotypes O1 and O139 are the causative agents of the severe diarrhoeal disease cholera. Non‐O1/non‐O139 serotypes are usually associated with less severe gastrointestinal infections as well as blood, wound and ear infections (Morris, 1990; Sack *et al*., 2004).

The ecology of environmental *V. cholerae* has been widely studied in (mostly tropical and subtropical) countries where cholera is endemic with focus on serotypes O1 and O139 (Huq *et al*., 2005; Alam *et al*., 2006a). In contrast, the ecology of non‐O1/non‐O139 populations in temperate climate zones is much less understood (Schuster *et al*., 2011), despite the fact, that global climate change scenarios are indicating increasing numbers of *Vibrio*‐associated diseases linked to increased water temperatures (Baker‐Austin *et al*., 2013; Vezzulli *et al*., 2013). A recent review has convincingly summarized and elaborated the specific environmental variables that explain variance in *V. cholerae* abundance on a global scale (Takemura *et al*., 2014) but did not differentiate between the climate zones. Moreover, these authors claimed that culture independent methods such as fluorescent *in situ* hybridization (FISH) or quantitative polymerase chain reaction (PCR) should be used as a basis of a ‘mass‐balanced’ approach to assess the preferred micro‐habitat of the Vibrios (Takemura *et al*., 2014). Up to now, reliable quantitative data on *V. cholerae* abundance in the environment are scarce.

In Austria, several ear, wound and blood infections caused by *V. cholerae* non‐O1/non‐O139 have been documented in the past decade (Huhulescu *et al*., 2007) that could be related to recreational activities in the large saline lake Neusiedler See. Subsequently, the autochthonous existence of *V. cholerae* non‐O1/non‐O139 strains in the lake could be demonstrated (Kirschner *et al*., 2008).

In order to elucidate the environmental predictors of *V. cholerae* and to allow a first estimation of the health risk for visitors of the lake on the basis of solid quantitative data, we applied a recently developed new protocol for the rapid and sensitive quantification of *V. cholerae* in water samples with catalysed reporter deposition (CARD)‐FISH in combination with solid‐phase cytometry (SPC) (Schauer *et al*., 2012). Simultaneously, *V. cholerae* attached to crustacean zooplankton was determined via FISH in combination with epifluorescence microscopy (EFM/FISH) (Kirschner *et al*., 2011). Water samples and crustacean zooplankton were analysed with the FISH methods in comparison to a cultivation based (membrane filtration) approach at five stations of Lake Neusiedler See, Austria, and three selected adjacent smaller alkaline lakes over a period of 20 months. Via multivariate statistical analysis the main predictors of *V. cholerae* abundance were identified. An estimation of the health risk was achieved by comparing the measured *V. cholerae* concentrations with infective dose values from the literature.

## Results

Water and zooplankton was analysed for samples taken at five stations in Neusiedler See as well as in three selected shallow alkaline lakes nearby (Fig. S1). Of the shallow alkaline lakes, the Zicklacke and the Oberstinker were sampled over the whole period; the Unterstinker was only sampled in 2012.

### Quantification of *V* *. cholerae* in water

**Figure 1:**
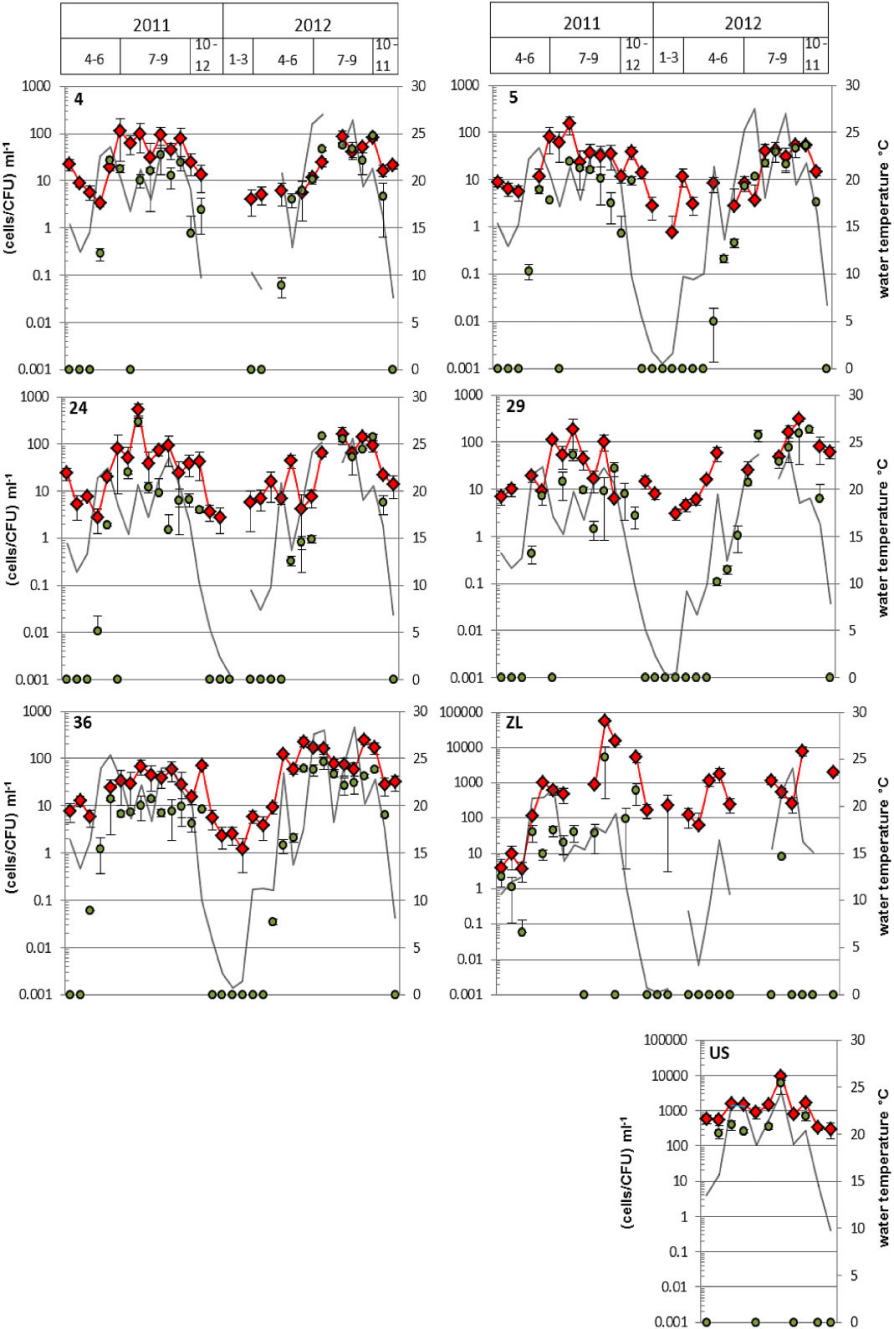
Concentrations of *V* *. cholerae* determined via SPC/CARD‐FISH (red diamonds) and the cultivation‐based method (green circles). Grey lines indicate the water temperature. Sampling points 4 to 36 represent the Neusiedler See, ZL the Zicklacke and US the Unterstinker. A missing water temperature value means that no sampling has taken place at that particular sampling point at that particular sampling time. If water temperature was measured, missing concentration values mean that samples could not be evaluated (e.g. due to high‐fluorescence background). Due to logarithmic scaling, a value of 0.001 means that no cells were detected. Values and error bars represent mean of three (cultivation) and three to eight (SPC/CARD‐FISH) replicates ± standard deviation. Values below 2 cells per ml (lake Neusiedler See) of SPC/CARD‐FISH, and below four cells per ml (soda lakes) are below the limit of quantification, and the presented values have to be taken as a qualitative detection.

Over all, *V. cholerae* numbers were significantly higher in the shallow alkaline lakes in comparison to the Neusiedler See [analysis of variance (ANOVA), *P* < 0.001]. *Vibrio cholerae* numbers determined with the SPC/CARD‐FISH approach ranged between < 2 (lowest limit of quantification) and 550 (±160) cells ml^−1^ for the lake and between < 4 (lowest limit of quantification) and 56 300 (±11,200) cells ml^−1^ for the shallow alkaline lakes (Fig. 1). *Vibrio cholerae* numbers obtained from cultivation ranged between 0 and 300 (±60) CFU ml^−1^ for the lake and between 0 and 6100 (±3200) CFU ml^−1^ for the shallow alkaline lakes (Fig. 1).

For each sampling point, a clear seasonal cycle could be observed. No significant differences between the sites were found (ANOVA, *P* > 0.1). During the winter season, when water temperature fell below 10°C, *V. cholerae* could not be cultivated. In contrast, with SPC/CARD‐FISH, we were able to reliably determine *V. cholerae* concentrations. During this period, cell numbers ranged between < 2 and 60 (±20) cells ml^−1^ for the lake and between < 4 and 2000 (±300) cells ml^−1^ for the Zicklacke. Highest concentrations were found with both methods when water temperature was between 20°C and 25°C, except for sampling point 36, where the highest numbers were observed above 25°C. During the summer periods (May–October) results obtained via cultivation were significantly lower than SPC/CARD‐FISH (Wilcoxon test; *P* < 0.001; median = 10 CFU ml^−1^ versus 45 cells ml^−1^). Nevertheless, when looking at data from summer 2012 only, *V. cholerae* concentrations at sampling points 4, 5, 24 and 29 were not significantly different between the two methods (*P* > 0.05). Moreover, a highly significant correlation between the results obtained by the two methods was observed when pooling data from all sampling points for the summer periods of both years (Spearman's rho = 0.694; *P* < 0.001; *n* = 141). When comparing data from summer 2011 with summer 2012 (inter‐annual comparison), sampling point 36 showed with both methods significantly higher values in 2012 [ANOVA; colony‐forming units (cfu): *P* < 0.05, median summer 2011 = 8 CFU ml^−1^, median summer 2012 = 29 cfu ml^−1^; SPC: *P* < 0.005, median summer 2011 = 36 cells ml^−1^, median summer 2012 = 76 ml^−1^]. To find an explanation for this difference, we tested for significant differences for all environmental parameters between the 2 years. There was a general trend at this site for nearly all parameters towards higher values in 2012, mainly due to a ‘concentration effect’ with significant differences (*P* < 0.05 to < 0.001) for pH, alkalinity, conductivity, dissolved organic carbon, total cell numbers and especially ammonium, which tripled in 2012 (52 μg/L) in comparison to 2011 (15 μg/L). This ‘concentration effect’ was due to a hotter summer 2012 with significantly higher maximum air and water temperatures (28.3°C in 2012 compared with 25.4°C in 2011) and lower water depth of this shallow lake. Why this increase in *V. cholerae* abundance was only observed at site 36 may be explained by the specific hydro‐morphological conditions of this sampling site. While site 36 is completely surrounded by reed and sheltered from winds, all other sampling sites are ‘open water’ sites and exposed to wind and wind‐induced homogenization of the water column. Indeed, there were fewer significant differences between the 2 years for the other ‘open water’ sites (e.g. no difference in pH, total bacterial numbers; lower differences found for DOC and ammonium concentrations). So, the concentration effect and higher maximum temperatures may have a more pronounced effect at site 36 than at the other sites.

In the shallow alkaline lake Zicklacke, with one exception, no culturable *V. cholerae* could be detected in 2012, while in 2011 culturable *V. cholerae* were observed throughout the warm period. For the Oberstinker – the shallow lake reaching the highest pH and salinity values of all alkaline lakes in the region (Kirschner *et al*., 2002) – it was not possible to detect any *V. cholerae* via SPC/CARD‐FISH during the whole investigation period. At most occasions, fluorescence background levels were too high to enable analysis with the SPC, even with 10 μl sample volume, and in the few samples where background fluorescence was acceptable, no *V. cholerae* cells were found. Coincidentally, we were not able to cultivate any *V. cholerae* from this lake. Growth of yellow colonies on thiosulfate citrate bile sucrose (TCBS) agar was observed, but those colonies failed to grow on nutrient agar without sodium chloride, they were negative by ompW‐PCR and were therefore not considered as *V. cholerae*.

### Quantification of *V* *. cholerae* on zooplankton

**Figure 2:**
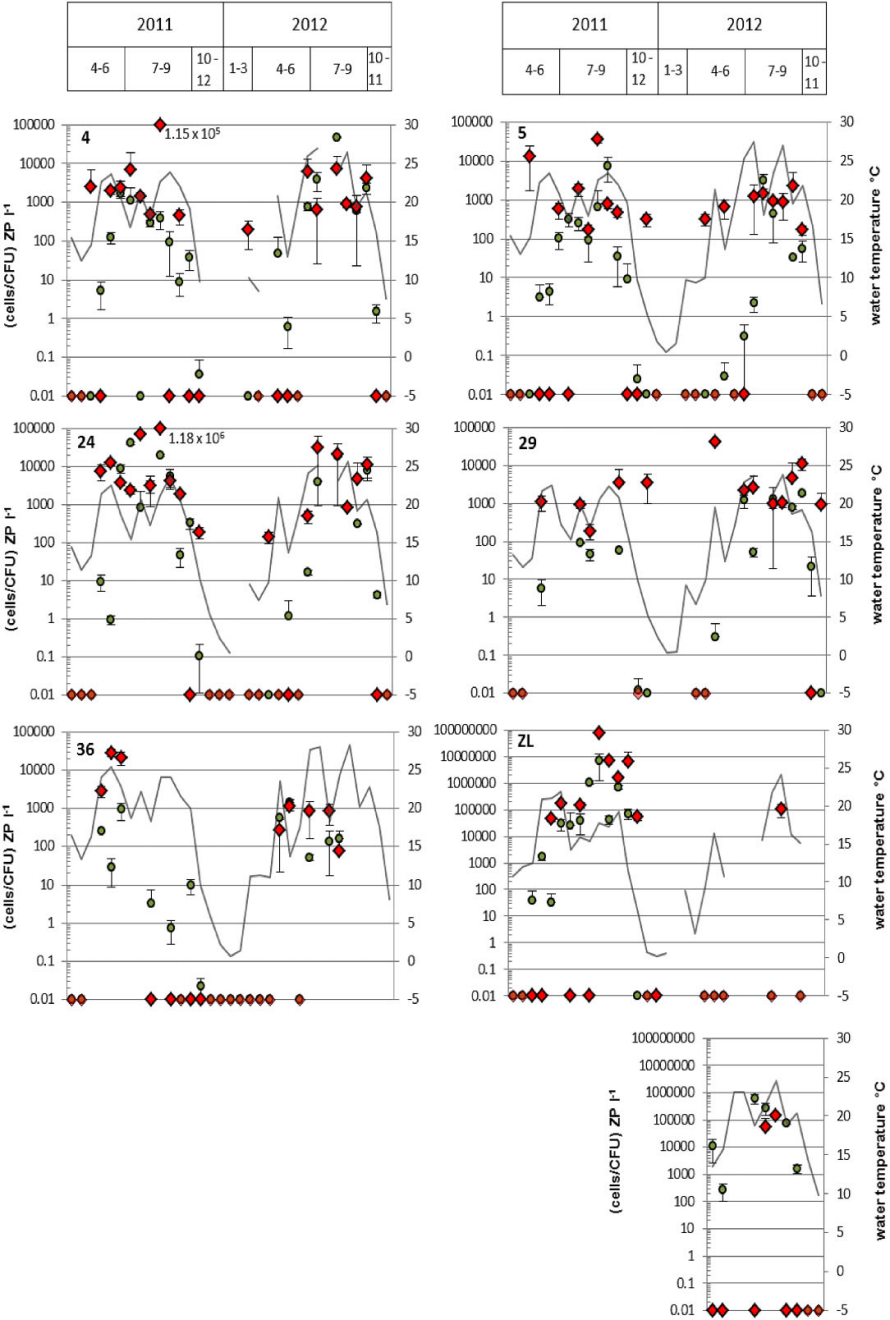
Concentrations of *V* *. cholerae* associated with crustacean zooplankton determined via EFM/FISH (red diamonds) and the cultivation‐based method (green circles). Grey lines indicate the water temperature. Sampling points 4 to 36 represent the Neusiedler See, ZL the Zicklacke and US the Unterstinker. A missing water temperature value means that no sampling has taken place at that particular sampling point at that particular sampling time. If water temperature was measured, missing concentration values mean that samples could not be evaluated (e.g. because of too low zooplankton abundance). Due to logarithmic scaling, a value of 0.01 means that no cells were detected. Values and error bars represent mean of three replicates ± standard deviation (cultivation and EFM/FISH).

**Figure 3:**
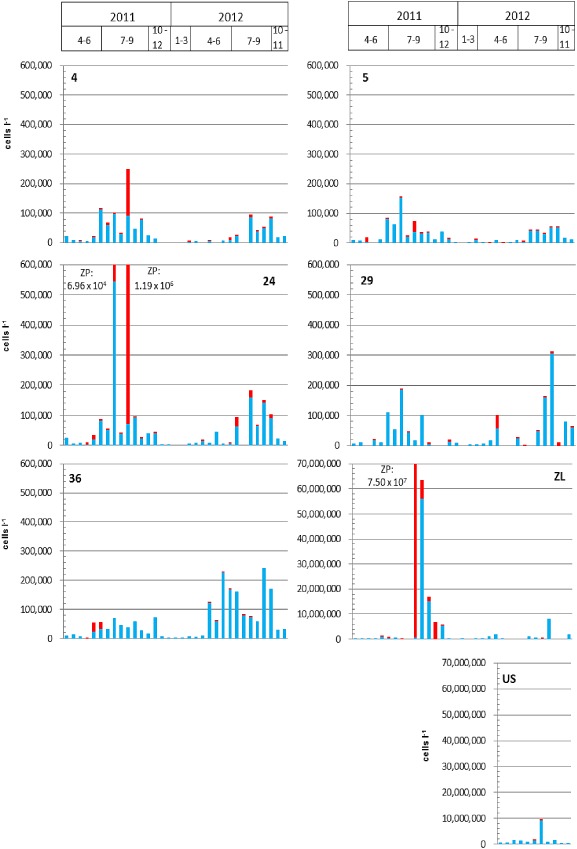
Total concentration of *V* *. cholerae* (in cells per litre) determined via SPC/CARD‐FISH in the water (blue columns) and via EFM/FISH on crustacean zooplankton (red columns). Sampling points: 4 to 36 represent the Neusiedler See, Zicklacke (ZL) and Unter Stinker (US).

Epifluorescence microscopy/FISH was selected for quantification of *V. cholerae* associated with crustacean zooplankton because zooplankton‐associated cells showed sufficient fluorescence intensity with FISH probes, and this procedure was quicker than the SPC/CARD‐FISH approach due to a shorter hybridization time. Over all, zooplankton‐associated *V. cholerae* numbers determined via EFM/FISH ranged from 0 to 1.18 × 10^6^ cells L^−1^ for the lake and between 0 and 7.5 × 10^7^ cells L^−1^ for the shallow alkaline lakes (Fig. 2). Concentrations obtained by cultivation ranged from 0 to 4.6 × 10^4^ CFU L^−1^ for the lake and from 0 to 6.8 × 10^6^ CFU L^−1^ for the shallow alkaline lakes (Fig. 2). Results from both methods were significantly intercorrelated (rho = 0.601; *P* < 0.001; *n* = 156). High cell numbers of *V. cholerae* associated with crustacean zooplankton were found only during short periods in summer (Fig. 3) when temperature was high and cladocerans were a dominating zooplankton group (Fig. S2). Highest average *V. cholerae* cell numbers that were attached to one zooplankton individual were 3.6 × 10^4^ and 8.6 × 10^4^ cells for the lake and the shallow saline lakes respectively. For both methods, *V. cholerae* abundances were positively correlated with cladoceran numbers (for cultivation: rho = 0.452, *P* < 0.001, *n* = 156; for EFM/FISH: rho = 0.301, *P* < 0.001, *n* = 161), while no significant correlation with copepods was found.

### Relation to environmental variables

Table 1 provides an overview of the basic ecological characteristics of the eight sampling sites. The shallow alkaline lakes differ significantly from the Neusiedler See (sampling points 4 to 36). All measured parameters, except oxygen (and NH_4_ and NO_3_ that were only determined in the Neusiedler See), exhibit much higher values in the shallow alkaline lakes. Within the five sampling points of the Neusiedler See, sampling point 29 (near to the shoreline and close to the run‐off from a sewage treatment plant) and 36 (within the reed belt) show significant differences for several environmental variables compared with the other three sampling points (ANOVA; Tukey‐Honestly‐Significant‐Difference post‐hoc comparison; *P* < 0.05 to < 0.001). The saline water at 29 is diluted with the freshwater entering the lake leading to lower pH, conductivity and alkalinity values. Significantly less zooplankton and lower oxygen values are found there, while nutrient concentrations (P_tot_ and NH_4_ values) are significantly higher. As sampling point 36 is completely surrounded by dense reed vegetation, concentrations of suspended solids are significantly lower (due to less wind induced sediment re‐suspension), while DOC values are significantly higher originating from refractory dissolved organic matter of decaying *Phragmites australis* plants (brownish water colour). In addition, significantly higher alkalinity and conductivity values are observed than in the open lake stations 4, 5 and 24. At station 24, significantly higher zooplankton (copepods and cladocerans) are observed.

**Table 1:** Ecological characteristics of the different sampling points. Data represent mean values and minimum and maximum values in brackets. DOC, dissolved organic carbon; Ptot, total phosphorus; TSS, total suspended solids; Chla, chlorophyll a; TBN, total bacterial numbers

Sampling point	pH	Conductivity	Carb.Alkalinity	DOC	P_tot_	O_2_	TSS	Chl a	NH_4_‐N	NO_3_‐N	Copepods	Cladocerans	Biomass	TBN
		μS cm^−1^	mmol l^−1^	mg l^−1^	μg l^−1^	mg l^−1^	mg l^−1^	μg l^−1^	μg l^−1^	μg l^−1^	Ind l^−1^	Ind l^−1^	μg DW l^−1^	cells ml^−1^
**4**	8.8	2100	9.92	16.5	45	9.4	64.6	8.4	14	66	7.1	11.7	68	8.80 × 10^6^
	(8.7–8.9)	(1700–3100)	(8.83–11.86)	(9.3–19.2)	(16–132)	(8–11.7)	(27.9–202)	(2.9–20.8)	(0–68)	(0–146)	(0.35–23.6)	(0.01–37.3)	(16–272)	(3.08 × 10^6^–1.56 × 10^7^)
**5**	8.8	1900	9.03	13.7	66	9.9	87.0	13.5	9	75	4.9	9.6	55	8.40 × 10^6^
	(8.6–8.9)	(1400–3000)	(7.85–10.99)	(12–15.7)	(6–196)	(8–17.5)	(12.2–327)	(3.7–30.5)	(0 43)	(0–204)	(0.52–12.1)	(0.01–43.3)	(3–202)	(3.35 × 10^6^–1.58 × 10^7^)
**24**	8.8	1900	8.64	13.9	53	9.9	70.3	11.1	7	89	9.8	10.5	79	9.14 × 10^6^
	(8.7–8.9)	(1400–3100)	(7.44–10.93)	(11.4–17.6)	(18–166)	(8.1–14.5)	(18.1–202)	(3.8–29.4)	(0–43)	(0–218)	(0.60–32.1)	(0.00–60.6)	(13–307)	(1.46 × 10^6^–1.58 × 10^7^)
**29**	8.3	1600	7.31	11.4	103	7.3	53.4	9.6	70	83	3.4	3.1	26	8.77 × 10^6^
	(7.5–8.9)	(1100–2600)	(4.99–9.19)	(5.8–17.4)	(29–297)	(0.6–16.7)	(12.3–202)	(1.8–22.7)	(0–373)	(0–238)	(0.08–21.5)	(0.00–48.6)	(2–177)	(2.27 × 10^6^–2.45 × 10^7^)
**36**	8.5	2200	11.08	22.7	36	8.7	20.0	7.9	27	86	8.8	6.6	73	1.15 × 10^7^
	(8.3–8.8)	(1600–3100)	(8.89–12.97)	(10.3–35.8)	(23–64)	(5.7–11.7)	(10.8–88.7)	(3.1–17.5)	(1–99)	(10–146)	(0.07–49.6)	(0.20–32.4)	(1–298)	(3.13 × 10^6^–2.95 × 10^7^)
**ZL**	9.6	11000	50.66	179	1230	8.1	4710	180			586	153	8210	1.09 × 10^8^
	(9.1–10.4)	(400–35700)	(6.22–144.6)	(9.1–462)	(106–3530)	(1.9–16.8)	(5–75300)	(0.2–723)			(0.00–3670)	(0.00–1070)	(2–56200)	(3.75 × 10^6^–3.29 × 10^8^)
**US**	9.8	6800	53.15	101	505	7.7	776	89.5			500	85	3360	1.27 × 10^8^
	(9.6–10.0)	(5000–8800)	(37.92–70.54)	(62.3–158)	(184–1070)	(4.9–13.0)	(165–2270)	(7.4–172)			(110–995)	(0.00–260)	(1250–6200)	(7.45 × 10^7^–2.65 × 10^8^)
**OS**	9.9	13000	97.46	87.3	5600	9.7	1480	51.8			420	201	3340	8.26 × 10^7^
	(9.2–10.4)	(900–29600)	(2.35–239.8)	(8.6–180)	(600–16900)	(6.4–15.6)	(52–5700)	(5.8–111)			(0.00–1700)	(0.00–1175)	(18–6700)	(2.75 × 10^7^–2.48 × 10^8^)

Two major groups of crustacean zooplankton > 250 μm were observed. In the Neusiedler See cladocerans (predominantly *Diaphanosoma mongolianum*) dominate during the warm period at most stations (maximum of 60 indiv. per L, point 24), while copepods (predominantly *Arctodiaptomus spinosus*) are present over the whole year, with highest concentrations even in winter (point 36; 50 indiv. per L; Fig. S3). With a few exceptions, very low zooplankton concentrations were observed at sampling point 29, at point 36 high zooplankton concentrations only occurred from October to June. Zooplankton concentrations in Zicklacke (ZL) and Unterstinker (US) were two orders of magnitude higher than in the lake with up to 3700 indiv. per L.

By using a generalized estimating equation model (GEE), the main environmental predictors of the *V. cholerae* abundance in the Neusiedler See and the shallow alkaline lakes were extracted from the data set (list of parameters fed into the models are listed in Table S1). For this purpose only the abundance data determined with the SPC/CARD‐FISH method (log transformed for achieving a symmetric distribution) was used, due to the fact that at many time points *V. cholerae* were not culturable. In the model of the Neusiedler See, water temperature, Cladoceran abundance, total suspended solids and ammonium levels were positive predictors on *V. cholerae* abundance (Table 2A). Conversely, copepod abundance was negatively related. From the standardized model coefficients, the relative impact of the predictor variable can be deduced. Water temperature and total suspended solids showed the highest values and were thus the strongest predictors of *V. cholerae* abundance in the Neusiedler See. For the shallow alkaline lakes, the model contained water temperature, DOC and P_tot_ as positive predictor variables. In addition, conductivity was negatively related to *V. cholerae* abundance (Table 2B). According to the standardized model coefficients, DOC and conductivity were the strongest predictors of *V. cholerae* abundance in the alkaline lakes. For a comparison, the robust model for the Neusiedler See was also adopted to the alkaline lakes (Table 2A) and vice versa (Table 2B). In both cases, the ‘adopted’ model was less appropriate to predict *V. cholerae* abundance with higher *P*‐values for some of the predictor variables included in the model.

**Table 2:** Results of parameter estimates based on the final robust generalized estimating equations (GEE) model of *V* *. cholerae* abundance in the lake Neusiedler See and in the shallow alkaline lakes. (A) Robust model calculated for the Neusiedler See that was comparatively applied to the alkaline lakes. (B) Robust model calculated for the alkaline lakes that were comparatively applied to the Neusiedler See. All predictors log transformed. Standardized model coefficient calculated as model coefficient times standard deviation of the respective parameter. Generalized estimating equations model with normal deviates and identity link, sampling point as subject effect, sampling time point as inner subject effect and a two‐dependent correlation structure. n.a., not analysed (no data available)

A:		Robust model Neusiedler See				Alkaline lakes	
Variable	Model coefficient	95% confidence interval	Standardized model coefficient	Wald Chi^2^	*P*‐value	Model coefficient	*P*‐value
Constant term	0.15	−0.28–0.58		0.5	>0.05	1.26	<0.001
Water temperature [°C]	0.69	0.55–0.84	0.154	85.1	<0.001	1.03	<0.001
Copepod abundance [indiv. m^−3^]	−0.19	−0.28–−0.09	−0.099	51.1	<0.001	−0.15	<0.001
Cladoceran abundance [indiv. m^−3^]	0.07	0.05–0.09	0.071	16.1	<0.001	0.05	<0.001
Total suspended solids [mg L^−1^]	0.45	0.15–0.75	0.320	8.8	<0.005	0.21	>0.05
NH_4_ [μg L^−1^]	0.10	0.04–0.16	0.029	9.4	<0.005	n.a.	n.a.

B:		Robust model alkaline lakes				Neusiedler See	
Variable	Model coefficient	95% confidence interval	Standardized model coefficient	Wald Chi^2^	*P*‐value	Model coefficient	*P*‐value
Constant term	3.24	3.06–3.42		1279.1	<0.001	1.63	>0.05
Water temperature [°C]	1.59	1.42–1.76	0.511	327.5	<0.001	0.58	<0.001
Dissolved organic carbon [mg L−^1^]	2.80	2.53–3.08	1.090	396.1	<0.001	1.33	<0.01
Total phosphorus [μg L−^1^]	1.03	0.56–1.50	0.469	18.4	<0.001	0.41	<0.05
Conductivity [μS cm−^1^]	−2.80	−3.23–−2.38	0.964	165.9	<0.001	−0.99	>0.05

## Discussion

By applying the recently developed cell‐based SPC/CARD‐FISH approach for quantification of *V. cholerae* (Schauer *et al*., 2012) on a large temporal and spatial scale, we could clearly demonstrate the usefulness of the new method for the elucidation of the main environmental predictors of *V. cholerae* abundance and for estimating the health risk for visitors of the lake. Despite of the high turbidity of the water samples, *V. cholerae* could be reliably quantified at five stations in the Neusiedler See and two adjacent shallow alkaline lakes over a period of 20 months. However, the CARD‐FISH/SPC approach is limited to the detection/quantification of bacterial targets that are present at sufficiently high numbers (at least between 2 and 80 cells per ml). Due to its methodical elaborateness, it can be recommended for scientific studies of the ecology of pathogens or for risk assessment, but – at present – not for routine application. *Vibrio cholerae* cell numbers determined via SPC/CARD‐FISH were equal (2012) or higher (2011) than cultivation‐based numbers, and both methods were significantly intercorrelated. Possible reasons for differences between the two methods have been discussed in detail in Schauer and colleagues (2012). The reason why cultivation led to equal results as SPC/CARD‐FISH in 2012 at the three sampling sites 4, 5 and 24 was most likely the significantly higher average and maximum water temperature in 2012, leading to a higher ratio of culturable cells.

### Environmental predictors of *V* *. cholerae* abundance

#### Neusiedler See

A combination of environmental variables obviously had a significant influence on *V. cholerae* concentrations in Neusiedler See. With the generalized estimation equation model water temperature, Cladoceran abundance, total suspended solids and ammonium levels were extracted as positive predictors. In contrast, copepod abundance was negatively related to *V. cholerae* abundance.

It has been shown that temperature is a main driver of *V. cholerae* growth in many ecosystems (Takemura *et al*., 2014) and that *V. cholerae* cannot be detected (at least by culture based methods) at temperatures below ∼10°C. In Neusiedler See and the shallow saline lakes, highest numbers of *V. cholerae* were found at temperatures above 20°C at all sampling sites. However, in contrast to the culture‐based method, we were able to quantify *V. cholerae* also during the winter season in the open water column of the lake with the SPC/CARD‐FISH method. Even though cell numbers of *V. cholerae* were not very high, the absence of cfu suggests that viable but non‐culturable *V. cholerae* may be present during winter in the planktonic state. Other reports have suggested that *V. cholerae* might overcome cold seasons in the sediment (Vezzulli *et al*., 2009) or on chitinous surfaces with cryoprotective effect (Amako *et al*., 1987). We did not find significant amounts of *V. cholerae* on crustacean zooplankton in winter, probably because only copepods were present, that were shown to have a negative effect on *V. cholerae* proliferation in the lab (Kirschner *et al*., 2011).

When comparing the abundance of *V. cholerae* on crustacean zooplankton in comparison to the water column, it became evident that at all stations in the Neusiedler See (and in the shallow alkaline lakes) the majority of *V. cholerae* cells were found in the water samples and not on crustacean zooplankton (Fig. 3; cell numbers related to 1 L of water). Only at very few occasions during the warm period the crustacean zooplankton was the predominant (16 August 2011 at sampling points 4 and 24; 2 July and 10 September at sampling point 29) or an important habitat for *V. cholerae* (2 May 2012 at sampling point 29 and 23 May, 6 June, 21 June 2011 at sampling points 4 and 36) (Fig. 3). During the warm period, cladocerans usually contribute to a large extent to the total crustacean zooplankton abundance in Neusiedler See (Fig. S2). The dominant cladoceran species in the lake, *Diaphanosoma mongolianum*, was recently demonstrated to have a stimulating effect on *V. cholerae* proliferation in lab experiments (Kirschner *et al*., 2011) and a significant positive correlation between *V. cholerae* and cladocerans was also observed in this study (rho = 0.319; *P* < 0.001; *n* = 130). In contrast, the copepods *Arctodiaptomus spinosus* had a negative effect on *V. cholerae* in lab experiments (Kirschner *et al*., 2011) and showed a negative correlation to *V. cholerae* in this study (rho = −0.237; *P* < 0.01; *n* = 127). These differential effects that different zooplankton species were shown to exert on *V. cholerae* have now been corroborated with the analysis of a large environmental data set. In general, crustacean zooplankton was shown to be the dominant reservoir for environmental *V. cholerae* in many studies, mainly from marine and estuarine environments or brackish water habitats (Huq *et al*., 1983; Tamplin *et al*., 1990). In comparison to those environments the water of the Neusiedler See is special as it offers perfect conditions for *V. cholerae*, with a pH ∼8.8, a salinity of ∼0.2% and high organic matter concentrations, which may be the reasons for their effective growth in the water column (Kirschner *et al*., 2008).

Another positive predictor of *V. cholerae* abundance was the concentration of total suspended solids (TSS). Sediments have been shown to serve as a sink for *V. cholerae* (Hood and Ness, 1982; Vezzulli *et al*., 2009) and may pose a threat to water quality when re‐suspended into the water column. We thus checked in 2011 by means of a cultivation‐based five‐replicate most probable number (MPN) assay, whether the sediment of the lake is a possible sink for *V. cholerae* (see Fig. S3 for detailed results and method description). Over the year 2011, maximum concentrations of 1.6 × 10^4^ MPN g^−1^ were determined. At TSS concentrations of 200 mg L^−1^, as they are frequently observed in the lake due to wind induced re‐suspension, 3.2 × 10^3^ MPN per L may be maximally re‐suspended from the sediment. Due to the different methods used (membrane filtration and SPC/CARD‐FISH for the water column and MPN approach for the sediments), a direct quantitative comparison is not possible. However, the numbers indicate that the sediment may be a substantial source of the total *V. cholerae* abundance in the water.

Surprisingly, ammonium levels were found to be positively related to *V. cholerae* concentrations in the lake. No such positive relationship has been reported in the literature so far. In contrast, a negative correlation was found in Blackwell and Oliver (Blackwell and Oliver, 2008), and lake‐specific characteristics may be responsible for this observation.

#### Shallow alkaline lakes

With the exception of temperature, other factors than in the lake were significant predictors of *V. cholerae* abundance. A significant negative relation with conductivity was observed. During summer, these shallow lakes usually dry up completely and are refilled with water from precipitation over the winter season (Eiler *et al*., 2003). During the process of drying up, salinity, alkalinity and pH are steadily increasing transforming these shallow water bodies from ideal reservoirs of *V. cholerae* with concentrations up to 56 × 10^6^ cells per L to hostile environments with pH values of > 10 and salinities of > 36 mS cm^−1^ with NaCO_3_^2−^ and NaHCO_3_ as the dominating anions. The high salt concentrations observed in the ZL in 2012 with conductivity values ranging from 13.2 mS cm^−1^ to 35.7 mS cm^−1^ and pH values between 9.6 and 10.1 may explain why no *V. cholerae* were found in this year. In contrast, conductivity values ranged from 3.3 mS cm^−1^ to 13.1 mS cm^−1^ in 2011, and massive blooms of *V. cholerae* were observed.

General nutrient parameters (DOC concentration and total phosphorus levels) were positive predictors of *V. cholerae* abundance in the shallow lakes. As the alkaline lakes are extremely eutrophic and they offer ideal conditions for *V. cholerae* growth during the major part of the year (except during the last weeks of the drying‐up process), the cell numbers were approximately up to 100‐fold higher than in the Neusiedler See and can thus be denoted as hot spots of *V. cholerae*. These concentrations are also approximately one order of magnitude higher than the maximum concentrations (3 × 10^6^ – 6 × 10^6^ cells/cfu per L) observed so far for other ecosystems (Jiang and Fu, 2001; Heidelberg *et al*., 2002; Neogi *et al*., 2012).

Like in the Neusiedler See, also in the ZL the zooplankton was a hot spot of *V. cholerae* during a short period in summer 2011 (Fig. 3). A slightly significant correlation between *V. cholerae* and cladocerans was observed (rho = 0.419; *P* < 0.05; *n* = 31). Nevertheless, cladoceran abundance was not included in the final robust model as a positive predictor, because the other factors (nutrients, temperature and conductivity) obviously had a much more pronounced influence than the cladocerans.

### Estimation of public health risk

The Neusiedler See is intensively used for recreational activities throughout the year, most prominently for swimming (May–September) and surfing (throughout the year, unless the lake is covered with ice in January and February). Ear and wound infections due to *V. cholerae* nonO1/nonO139 infections have been reported for visitors of the lake since 2001 (Huhulescu *et al*., 2007). Up to now, no gastrointestinal infections have been reported. Maximum *V. cholerae* concentrations in the lake water observed in this study were 5.5 × 10^5^ per L. Adopting an infective dose of 10^5^ to 10^6^ cells from cholera studies with healthy volunteers (Sack *et al*., 1998; Cohen *et al*., 1999), one would have to swallow ∼200 ml to 2 L of water to acquire a gastrointestinal infection. The maximum number of *V. cholerae* cells found on a single Cladoceran zooplankton individual was 3.6 × 10^4^. At a maximum concentration of 60 Cladocerans per L, the amount of water necessary for a gastrointestinal infection would decrease significantly to ∼50 ml to 500 ml, volumes that are likely to be taken up by swimmers (Schets *et al*., 2011). For susceptible individuals, a few Cladoceran zooplankton organisms may thus be sufficient for infection, in a period of the year when they are full with *V. cholerae*.

There are no studies concerning the infective dose for ear or wound infections by *V. cholerae* but it can be assumed that it may be only a few cells, based on exponential ‘single‐hit’ models for other pathogens (Roser *et al*., 2014). Despite the fact that most of the infections related to the Neusiedler See occurred during summer, two persons were infected during a visit of the lake in November and December (Huhulescu *et al*., 2007), a period when water temperatures are below 10°C and *V. cholerae* are not culturable. With the CARD‐FISH/SPC protocol, however, we could demonstrate that *V. cholerae* cells were present at all sampling sites throughout the winter season, suggesting that viable but non‐culturable *V. cholerae* may cause infections in healthy humans.

## Conclusion

With the recently developed CARD‐FISH/SPC approach, we were able to detect and quantify *V. cholerae* in highly turbid aquatic environments where *V. cholerae* is an autochthonous component of the bacterial community. The results were significantly correlated to results from a cultivation‐based approach but showed mostly higher values. Especially in winter, *V. cholerae* could be quantified at all sites via SPC/CARD‐FISH, while cells were not culturable. With the developed data set, it was possible to elucidate the main environmental predictors of *V. cholerae* dynamics and to estimate the risk for persons visiting the lake for recreational reasons.

## Experimental procedures

### Sampling sites and sample collection

The Neusiedler See (47°42′N, 16°46′E) (Fig. S1) is the largest saline lake in Central Europe. It covers an area of approximately 320 km^2^ of which about 55% are covered by a dense reed belt accommodating several smaller open water bodies. The maximum depth is approximately 1.8 m. Five sites were selected for sampling to represent a wide range of ecological conditions: two open water sites, one in the northern (sampling point 24) and one in the southern part (sampling point 5) of the lake, one site situated within a small open water area within the reed belt (sampling point 36), one intermediate site (open water near to the reed belt, sampling point 4), and a site near to the shoreline (sampling point 29), close to the run‐off from a municipal sewage treatment plant. Three small shallow (maximum depth 0.5 m) alkaline lakes [Oberstinker (OS), Zicklacke (ZL) and Unterstinker (US)] situated along the eastern shore of the lake were also selected for sampling. Detailed information on the lake and on the alkaline lakes can be found elsewhere (Eiler *et al*., 2003; Kirschner *et al*., 2008). The Neusiedler See and the small alkaline lakes were monitored from April 2011 till November 2012, bi‐weekly during the summer (April till October) and monthly during the winter period (November till March). Samples for the US were taken only in 2012. All sampling at the Neusiedler See was performed from a motor boat; samples from the shallow lakes were taken by foot with appropriate chest waders. Sampling took place between 8 a.m. and 1 p.m. For quantification of *V. cholerae*, triplicate water samples were collected in clean, sterile, 500 ml glass flasks. Crustacean zooplankton samples were collected with vertical net hauls (mesh size, 250 μm). From each sampling point, eight zooplankton samples were taken and separately collected in clean 200 ml plastic bottles. Approximately 100–150 L of water was concentrated per bottle depending on the actual water depth. One zooplankton sample from each point was immediately fixed with 4% formaldehyde for quantification of crustacean zooplankton organisms. Water samples and the remaining zooplankton samples were transported in the dark at ambient temperature (Alam *et al*., 2006b) to the laboratory in Vienna within 1.5 h after the last sample was taken.

### Environmental parameters

Conductivity (LF 330, WTW, Germany), water temperature, pH (GHM, Seibold Vienna, Austria) and oxygen (OXI 330i, WTW) were measured *in situ*. For inorganic nutrients, chlorophyll a, TSS, total organic carbon (TOC) and DOC an extra water sample was collected in clean 1L plastic bottles and processed according to methods used in Eiler and colleagues (2003). Wind, air temperature and precipitation were measured on a daily basis at the Biological Research Institute Burgenland.

### Sample preparation

(i)  Water – Before analysis, water samples were pre‐filtered through a 250 μm mesh size net to exclude large particles (including crustacean zooplankton > 250 μm).
(ii)  Zooplankton – The total numbers of crustacean zooplankton > 250 μm, the distribution between copepods and cladocerans and their individual biomass were determined as previously described (Herzig, 1974). For quantification of *V. cholerae* associated with zooplankton, the samples were processed as follows (for details see Fig. S4): the collected zooplankton was carefully rinsed with excess sterile 1 × PBS to remove loosely associated bacteria and transferred to one sterile 50 ml tube for each sampling point. The supernatant was carefully removed with sterile Pasteur pipettes to a level, at which an even distribution of the zooplankton was still possible. For the detection of attached *V. cholerae* via FISH, three aliquots of ∼250 mg fresh weight (FW) of zooplankton were put into sterile 15 ml tubes. For cultivation, ∼1 g FW of zooplankton was put into a sterile micro‐mortar and homogenized with a sterile pestle (Radnoti; Monrovia, USA). Three aliquots of ∼250 mg FW of the homogenized zooplankton was put into sterile 1.7 ml tubes and treated with an ultrasonic water‐bath (Bandelin SONOREX; Berlin, Germany) for 20 min. For dry weight (DW) calculation ∼1 g FW of zooplankton was transferred into a 1.7 ml tube. The complete content was then filtered through a pre‐muffled glass microfiber filter (Ø 47 mm, Whatman), rinsed carefully with distilled water to remove salts form the PBS solution, dried for 48 h at 80°C in a muffle furnace (Nabertherm L40/11/B170, Lilienthal, Germany) and weighed. All weighing was done to the forth decimal place (0.1 mg) exactly. From the biomass, the dry weight values, and the dry weight to fresh weight ratio, the number of individuals used for the analysis of *V. cholerae* abundance on zooplankton was calculated according to the formula:
  $$\begin{array}{l} \text{Individuals per g FW}\\ \;=\text{DW}/\text{average individual biomass}\times \left(\text{DW}/\text{FW}\right) \end{array}$$
  Each replicate sample consisted of at least 35 and up to 1800 individuals.

### Quantification of *V* *. cholerae*

(i)  SPC/CARD‐FISH – For water samples, the newly developed SPC/CARD‐FISH protocol was applied as described in detail in Schauer and colleagues (2012). From the three replicate samples, three to eight subsamples of appropriate volume were taken, ranging from 50 μl to 2000 μl for the Neusiedler See and from 10 μl to 1000 μl for the alkaline lakes. Briefly, subsamples were fixed with paraformaldehyde (1% final concentration) and filtered on black polyester filters (CB04, AES – Chemunex). After hybridization with Vchomim1276‐HRP oligonucleotide probe, that is specific for *V. cholerae* and *V. mimicus* (Heidelberg *et al*., 2002), and tyramide‐based signal amplification, the membranes with the fluorescently labelled cells were analysed with SPC ChemScan RDI (AES‐Chemunex, Biomerieux, Ivry‐sur‐Seine, France). Details on the applied system have been described previously (Mignon‐Godefroy *et al*., 1997; Lemarchand *et al*., 2001).
(ii)  EFM/FISH – The triplicate subsamples of zooplankton were fixed with para‐formaldehyde (1% final conc) at 4°C. After overnight incubation, a tetrasodium‐pyrophosphate solution (0.01 M final concentration) was added and shaken on ice for another 6 h. Subsamples were sonicated for 3 min pulsed at 15 W (Branson digital sonifier 250D; CT, USA). Appropriate volumes were filtered on white polycarbonate membranes (Ø 25 mm, pore‐size 0.2 μm, Whatman) and air dried. FISH was carried out as previously described (Kirschner *et al*., 2011). Membranes were examined under a Nikon Eclipse 8000 microscope at ×1,250 magnification and 20 microscopic fields were evaluated.
(iii)  Cultivation – For water samples, cultivation was done by membrane filtration as previously described (Schauer *et al*., 2012). From each of the triplicate samples, subsamples of appropriate volume (1, 10, 100 ml for the Neusiedler See and 0.01, 0.1 and 1 ml, filled up to 10 ml with sterile deionized water for the shallow lakes) were taken, filtered and the membrane was placed on TCBS (Merck, Darmstadt, Germany) agar plates. For quantification of *V. cholerae* associated with zooplankton, 150 μl of a 10‐fold dilution series with 1 × PBS from the carefully prepared homogenized zooplankton were plated on TCBS agar plates. All TCBS agar plates were incubated for 18 h at 37°C. Yellow, flat, 1 to 3 mm diameter colonies were picked and processed as described in Schauer and colleagues (2012; Baron *et al*., 2007). Five representative presumptive isolates of each water and zooplankton sample were confirmed via species‐specific *ompW*‐based PCR (Schauer *et al*., 2012).

### Enumeration of total bacterial numbers

Total cell numbers were quantified via SYBR‐Gold (Invitrogen; CA, USA) staining. From each sampling site 4.75 ml of water was fixed with 0.25 ml 37% formaldehyde. One ml of an appropriate dilution of the fixed water sample (1:10 for samples from Neusiedler See; 1:100 for samples from the shallow alkaline lakes) was filtered on Anodisc filters (Ø 25 mm, pore‐size 0.2 μm, Whatman) and stained on a drop of SYBR‐Gold (Invitrogen, Lofer, Austria; 1:400× dilution of the stock) for 20 min. After drying, the filters were mounted on a microscopic slide under a drop of anti‐fading solution and analysed in a Nikon Eclipse 8000 microscope. Detailed information on the enumeration procedure can be found elsewhere (Riepl *et al*., 2011).

### Statistical analysis

Statistical analysis was performed with SPSS 22.0. For correlation analysis Spearman rank correlation was used. For testing differences between sites, ANOVA and Student's *t*‐test was applied. Parameters were log transformed to obtain normal distribution. To extract the main environmental predictors of *V. cholerae* abundance, we calculated two independent GEE models for the Neusiedler See and the shallow alkaline lakes. Generalized estimating equation was chosen, because this technique can handle parametric and non‐parametric variables simultaneously, and it provides reliable results even for data with correlated and not normally distributed residuals (Liang and Zeger, 1986). For the GEE models, sampling point was chosen as subject variable and the date of sampling as inner subject variable. The structure of the correlation matrix was defined as M‐dependent, with M = 2, meaning that samples taken at two consecutive sampling dates may be correlated due to their close temporal proximity. The model type was set to linear. In total, 18 environmental variables were put into the model as covariates (see Table S1) with potential influence on *V. cholerae* abundance. By step‐wise reducing the number of variables and removing intercorrelated variables as far as possible, two robust models with a combination of predictor variables influencing *V. cholerae* abundance in Lake Neusiedler See and the shallow alkaline lakes were generated.

## Supporting information


**Fig. S1.** Lake Neusiedler See, shared by Austria (A) and Hungary (H), and the shallow soda lakes Zicklacke (ZL), Unterstinker (US) and Oberstinker (OS) located along the eastern shore of the lake. The dark area of the Neusiedler See depicts the reed belt which makes up approximately 55% of the total lake area. For a representative sampling of the lake the following five sampling sites were chosen: two open water sites (5 in the South and 24 in the North), one point within the reed belt (36), one intermediate point (4) and one close to the run‐off from the only sewage treatment plant directly emitting into the lake (29).
**Fig. S2.** Seasonal variation of crustacean zooplankton abundance (individuals per litre) at the different sampling points. green bars: copepods; red bars: cladocerans). Sampling points 4 to 36 represent the Neusiedler See, ZL the Zicklacke and US the Unterstinker.
**Fig. S3.** Quantification of *V. cholerae* in sediment samples from the five different sampling points of the lake Neusiedler See via the MPN technique.
**Figure S4.** Experimental design for enumerating *V. cholerae* on zooplankton.
**Table S1.** Environmental variables used as potential predictors in the two GEE 1 models.
