# Supplementary material for: Dynamics of V ibrio cholerae abundance in Austrian saline lakes, assessed with quantitative solid‐phase cytometry
Source: Environ Microbiol. 2015 May 18;17(11):4366–78. doi: 10.1111/1462-2920.12861 (PMC4950322; doi:10.1111/1462-2920.12861)
Supplement: Supplementary file 1 — Fig. S1. Lake Neusiedler See, shared by Austria (A) and Hungary (H), and the shallow soda lakes Zicklacke (ZL), Unterstinker (US) and Oberstinker (OS) located along the eastern shore of the lake. The dark area of the Neusiedler See depicts the reed belt which makes up approximately 55% of the total lake area. For a representative sampling of the lake the following five sampling sites were chosen: two open water sites (5 in the South and 24 in the North), one point within the reed belt (36), one intermediate point (4) and one close to the run‐off from the only sewage treatment plant directly emitting into the lake (29). Fig. S2. Seasonal variation of crustacean zooplankton abundance (individuals per litre) at the different sampling points. green bars: copepods; red bars: cladocerans). Sampling points 4 to 36 represent the Neusiedler See, ZL the Zicklacke and US the Unterstinker. Fig. S3. Quantification of V. cholerae in sediment samples from the five different sampling points of the lake Neusiedler See via the MPN technique. Figure S4. Experimental design for enumerating V. cholerae on zooplankton. Table S1. Environmental variables used as potential predictors in the two GEE 1 models. [file EMI-17-4366-s001.pdf]

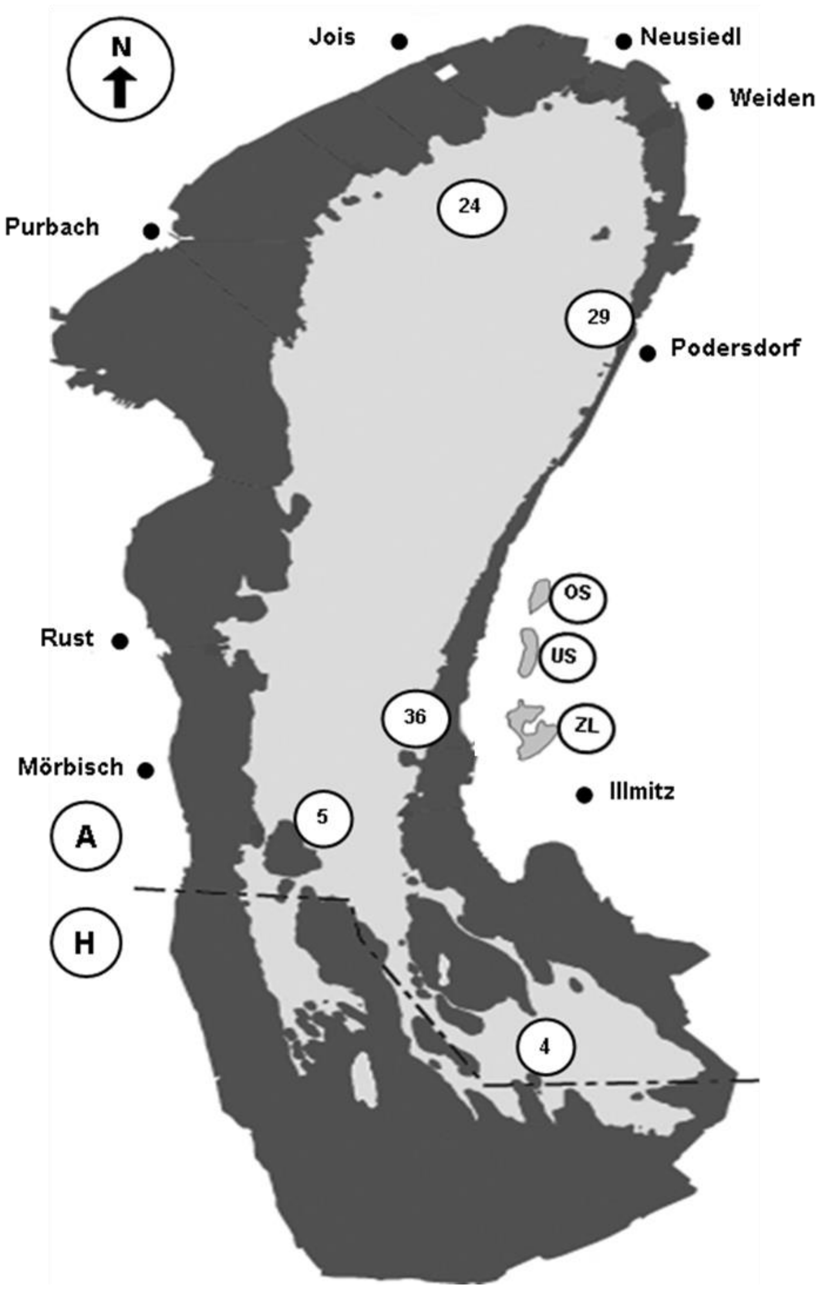

Fig. S1. Lake Neusiedler See, shared by Austria (A) and Hungary (H), and the shallow soda lakes Zicklacke (ZL), Unterstinker (US), and Oberstinker (OS) located along the eastern shore of the lake. The dark area of the Neusiedler See depicts the reed belt which makes up approx. 55% of the total lake area. For a representative sampling of the lake the following five sampling sites were chosen: two open water sites (5 in the South and 24 in the North), one point within the reed belt (36), one intermediate point (4) and one close to the run-off from the only sewage treatment plant directly emitting into the lake (29).

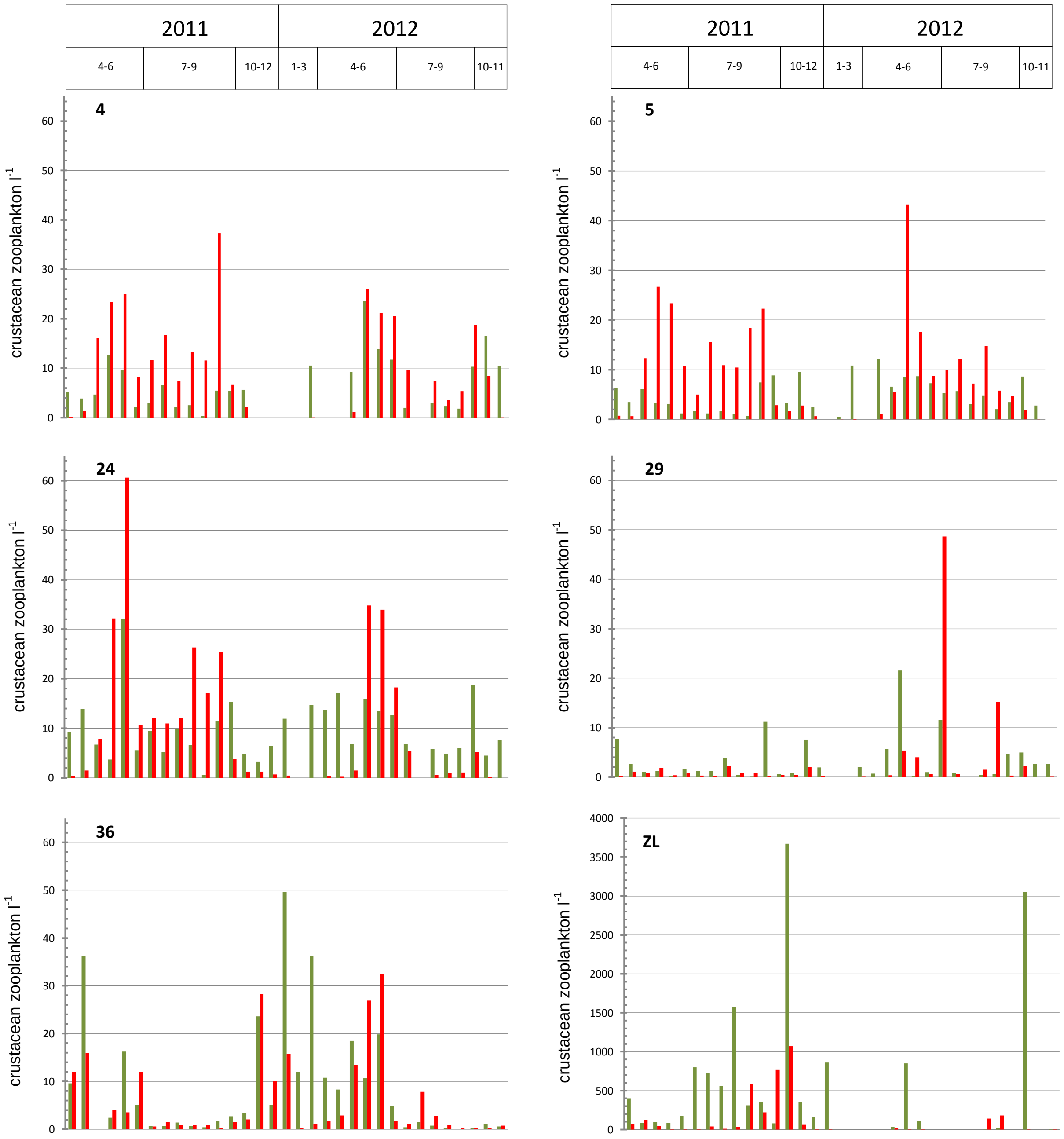

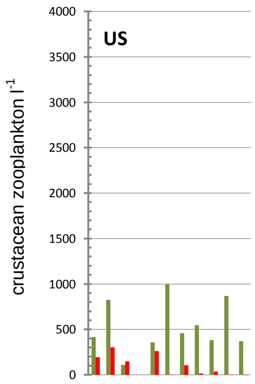

Fig. S2. Seasonal variation of crustacean zooplankton abundance (individuals per litre) at the different sampling points. green bars: copepods; red bars: cladocerans). Sampling points 4 to 36 represent the Neusiedler See, ZL the Zicklacke and US the Unterstinker.

Fig. S3. Quantification of *V. cholerae* in sediment samples from the 5 different sampling points of the lake Neusiedler See via the MPN-technique.

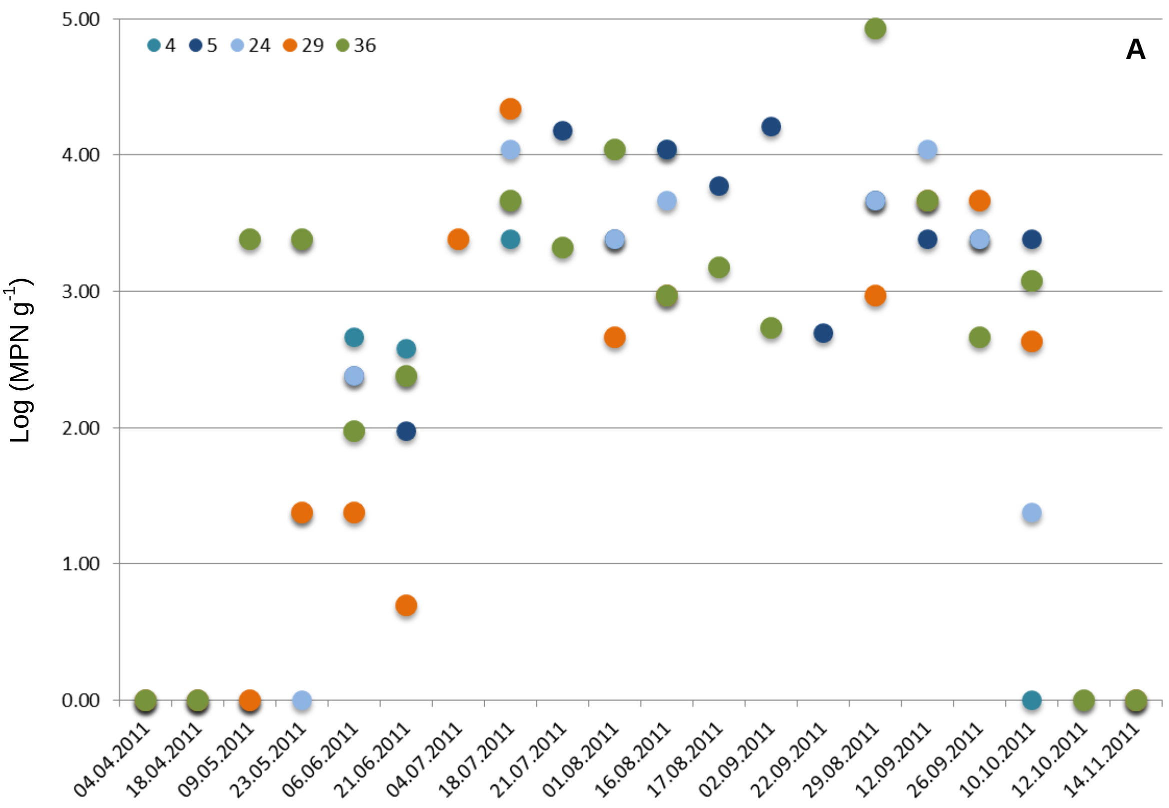

Quantification of *V. cholerae* in sediment samples

During the first year of monitoring, *V. cholerae* was also quantified in sediment samples from the lake and the shallow soda lakes via a culture based most-probable-number (MPN) technique. For this purpose 1 g of sediment was homogenized in 9 ml Ringer's solution. After homogenizing three more 1:10 dilutions were produced from the same suspension. A 5-replicate MPN was carried out with each of the four dilutions by adding 1 ml of diluted sediment sample to 9 ml enrichment medium (peptone water). Each subdivision was incubated for 18 to 24 h at 30°C. After incubation, each tube was observed for the presence or absence of growth and aliquots were streaked out on TCBS agar plates. After 24 h at 37°C presumptive *V. cholerae* colonies were transferred to nutrient agar without NaCl. When growth as observed, the isolates were tested with API 20E for their biochemical properties. Final MPN results were obtained from using a 5-tube MPN table.

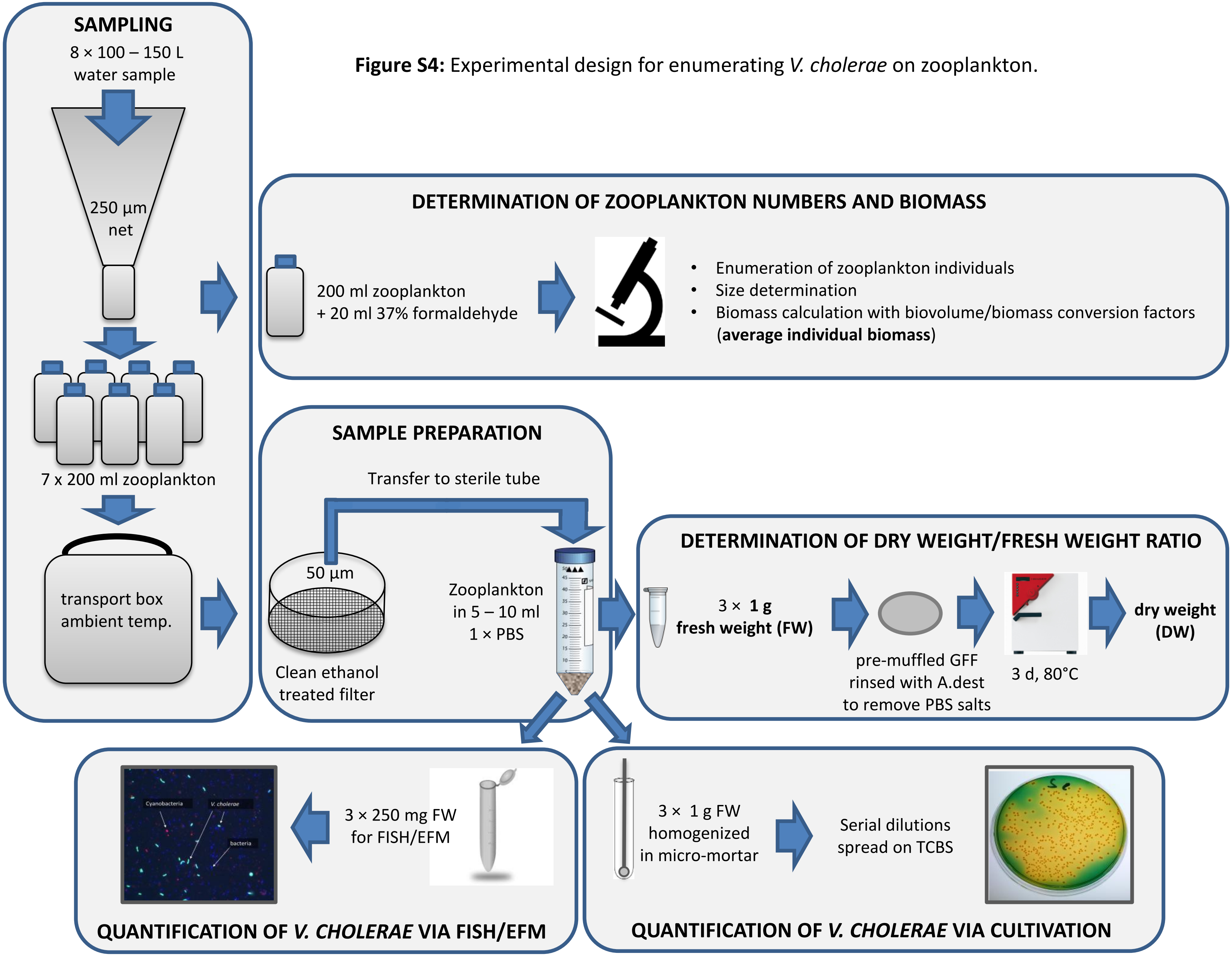

Figure S4: Experimental design for enumerating *V. cholerae* on zooplankton.

Table S1: Environmental variables used as potential predictors in the two GEE models:

variable	unit	transformation
Total bacterial cell numbers	Cells ml^{-1}	Log + 1
Copepod numbers	Individuals m^{-3}	Log + 1
Cladoceran numbers	Individuals m^{-3}	Log + 1
Water temperature	°C	no
Electrical conductivity	µS cm^{-1}	Log + 1
pH	---	no
Carbonate alkalinity	mmol L^{-1}	no
Dissolved organic carbon	mg L^{-1}	no
Total phosphorus	µg L^{-1}	Log + 1
Total suspended solids	mg L^{-1}	Log + 1
Humic ratio (254nm/365nm)	---	no
Oxygen saturation	%	no
NH_4	µg L^{-1}	Log + 1
NO_3	µg L^{-1}	Log + 1
Chlorophyll a	µg L^{-1}	Log + 1
Wind speed (average)	km h^{-1}	Log + 1
Wind speed (avg previous week)	km h^{-1}	Log + 1
Precipitation (sum previous week)	mm	no
